# It Is Useless to Resist: Biofilms in Metalworking Fluid Systems

**DOI:** 10.3390/life15060890

**Published:** 2025-05-30

**Authors:** Giulia von Känel, Lara Ylenia Steinmann, Britta Mauz, Robert Lukesch, Peter Küenzi

**Affiliations:** 1 Department of Microbiology, Blaser Swisslube AG, CH-3415 Hasle-Rüegsau, Switzerland; g.vonkaenel@blaser.com (G.v.K.); l.steinmann@blaser.com (L.Y.S.); r.lukesch@blaser.com (R.L.); 2 Department of Analytics, Blaser Swisslube AG, CH-3415 Hasle-Rüegsau, Switzerland; b.mauz@blaser.com

**Keywords:** metalworking fluids, amines, bacterial biofilms, biocides, biofouling, metagenomics, toxicity

## Abstract

Biofouling, the undesirable deposition of microorganisms on surfaces, is ubiquitous in aqueous systems. This is no different for systems running with water-miscible metalworking fluids (MWFs), which additionally contain many organic chemicals that create favorable conditions for growth and metabolism. Biofilm formation is thus inevitable, as there is no shortage of wetted surfaces in metalworking systems. MWF manufacturers tried in vain to offer resistance by using biocides and biostatic compounds as ingredients in concentrates and as tank-side additives. We report here that such elements, alone or as components of MWFs, did not prevent biofilm formation and had negligible effects on pre-established laboratory biofilms. Moreover, biofilms in metalworking systems are interwoven with residues, sediments, and metal swarfs generated during machining. Again, co-incubation of such “real” biofilms with MWFs had no significant effect on population size—but on population composition! The implications of this finding are unclear but could provide a starting point for the treatment of biofouling, as biofilm population structure might be of importance. Finally, we show that bacteria gain function in biofilms and that they were able to degrade a toxic amine in MWFs, which the same bacteria were unable to do in planktonic form.

## 1. Introduction

The concept of biofilms describes structured aggregates of microbial communities coated in self-produced extracellular polymeric substances comprised of extracellular DNA, polysaccharides, lipids, and proteins [1]. Such aggregates may appear as flocs in liquids or as slimy films, easily formed on wetted, inert, or living surfaces. They are assumed to be the predominant form of microbial life driving all major biogeochemical processes [2]. It is also understood that many biofilms form and/or expand due to exposure to external environmental cues, such as nutrient deficiency or excess, osmotic pressure, pH, oxidative stress, and antimicrobial agents [3,4,5,6,7]. Biofilms may consist of single or multiple species, confer many advantages on their inhabitants, and represent a much higher level of organization than single cells do.

Microbes have the capacity to attach to any given surface, especially if organic material had previously been deposited on it, such as carbohydrates, lipids, minerals, and/or calcium soaps, as is the case in industrial systems using water-miscible metalworking fluids (MWFs) [8]. MWFs are widely applied in metal removal and forming operations, dissipating heat and reducing friction. Three main categories of MWFs are utilized: emulsifiable oils, semisynthetics, and synthetics; all are formulated and sold as concentrates, containing anything from 10 to 20 organic ingredients. They are mixed at the end user’s site with water that subsequently accounts for 85% to 95% of the mixture [9]. The main components of MWF concentrates are mineral and ester oil, polyalphaolifins, or glycols. Emulsifiers, corrosion inhibitors, foam control agents, and lubricity enhancers are added as needed to enhance performance, stability, and functionality. Moreover, biocidal and biostatic components are added to some MWF formulations, as they are thought to keep microbiological agents under control [10]. However, regulatory pressure leading to significant restrictions on permitted concentrations is increasing, a trend that is expected to continue [11]. Basically, the range of potential components has narrowed down to isothiazolinones [8]: 2-methyl-2H-isothiazol-3-one (MIT), 1,2-benzisothiazol-3(2H)-one (BIT), and 5-chloro-2-methyl-4-isothiazolin-3-one (CMIT). The latter is mainly used as a tank-side additive, whereas the MIT and BIT are also used for in-drum conservation. Still, the high ratio of water and the evenly mixed-in organic components, aerated by recirculation, provide an appropriate base of life for planktonic bacteria, fungi, and archaea in all types of in-use as well as spent MWFs [8,12,13,14]. Significant, however, are the additional habitats on (machine) surfaces, which are wetted by contact with the coolant through flow, splashing, evaporation, and misting: Lines that supply and discharge the MWF from the site of action offer dozens of square meters of microbial settlement area. This is a considerable problem with single-filled machines, getting multiplied in centralized systems where the fluid is transported over long distances to and from many machining centers [15]. As in any aqueous system, biofilms are of far greater importance than their planktonic relatives, being the main cause for biofouling and microbiologically influenced corrosion [16,17,18].

Although biofilms in MWFs is a topic that receives a lot of attention in the industry [19], it is poorly documented in the published literature. Trafny and colleagues reported that the tetrazolium salt assay (MTT assay) could be efficiently used to assess the biofilm-forming capacity [20] and that biocides contained in the MWF formulation did not contribute to biofilm formation or control [21]. Other researchers found that NH_4_Cl and KH_2_PO_4_ negatively affect biofilm formation in MWFs when either present in excess or completely absent [22]. Finally, it was published that biofilms in MWFs were measurably reduced by successfully interfering with quorum sensing [23,24].

In this study, we report that bactericidal or bacteriostatic elements, as well as mineral oil-based and synthetic MWFs, respectively, blocked de novo biofilm formation but had largely negligible effects on established biofilms. Under some conditions, even biofilm growth was detected. However, depending on the type, MWFs had a detectable impact on the composition of the biofilm population, a process that is not yet understood. Moreover, we could show that biofilm populations developed capabilities that their planktonic relatives did not display, namely the ability to degrade an amine that was toxic to them.

## 2. Materials and Methods

### 2.1. Bacterial Species

*Pseudomonas oleovorans*, *Pseudomonas aeruginosa,* and *Morganella morganii* strains employed in all experiments were isolated from in-use MWF samples originating from Switzerland by standard heterotrophic plate count methods performed on Tryptone Soya Agar (TSA) prepared in-house (Oxoid CM0131, Thermo Fisher, Pratteln, Switzerland) at 40 g L^−1^. Incubation was performed at 35 °C for 2 to 3 days. Isolated bacterial species were subsequently identified by MALDI-TOF MS analysis of protein patterns at Mabritec AG (Riehen, Switzerland).

### 2.2. Biocides

For all experiments, technical standard biocides (bactericides to kill bacteria and fungicides to kill fungi) from industrial suppliers were used—the concentration indicated refers to a typical dose in a freshly prepared, 5% (*w*/*w*) in-use MWF: MIT (Acticide^®^ M 50; Thor GmbH, Speyer, Germany; active ingredient content: 50%) at a concentration of 100 ppm, BIT (PREVENTOL^®^ BIT 20 N; Lanxess Deutschland GmbH, Leverkusen, Germany; Active ingredient content: 20%) at a concentration of 150 ppm, and 2-n-octyl-4-isothiazolin-3-one (OIT; BIOBAN^TM^ O 45; Lanxess Deutschland GmbH, Leverkusen, Germany; active ingredient content: 45%) at a concentration of 50 ppm.

### 2.3. Metalworking Fluids

The described experiments were performed using two commercially available MWFs from Blaser Swisslube AG (Hasle-Rüegsau, Switzerland): the mineral oil-based, amine-free Blasocut 2000 CF (MWF M; Art. No. 00875-12) and the synthetic, amine-containing Grindex 10 CO (MWF S; Art. No. 01100-05). Both fluids were used in the formulation sold in 2023. Neither of the two concentrates contained listed bactericides [25].

For alkanolamine assays described in Section 2.7, MWF M was supplemented with 5% (*w*/*w*) 1-aminopropan-2-ol (MIPA; DOW Europe GmbH, Horgen, Switzerland) and 5% (*w*/*w*) 2,2′,2′′-Nitrilotri(ethan-1-ol) (TEA; DOW Europe GmbH, Horgen, Switzerland). Accordingly, the mineral oil content was reduced by 10% (*w*/*w*).

To prepare a 5% (*w*/*w*) working fluid, 47.5 g of sterile tap water was added to a sterile 100 mL beaker containing a sterile stirring bar before adding 2.5 g of concentrate. The mixture was stirred at 500 rpm for at least 30 min.

### 2.4. Mini “Microbial Evolution and Growth Arena” (MEGA) Experiments

TSA plates were prepared as described above, but shortly before solidifying, different concentrations of MIT (0, 100 ppm, 500 ppm, 2500 ppm), BIT (0, 150 ppm, 300 ppm, 600 ppm), MIPA (0, 0.15%, 0.3%, 0.6%) or TEA (0, 0.15%, 0.3%, 0.6%), were added and evenly mixed into the agar. In each case, 25 mL of the resulting mixtures were poured into sterile, 100 mm × 20 mm Petri dishes (#430167, Corning Inc., Corning, NY, USA) and left to cool completely. The resulting nutrient media was then cut along the diameter and parallel to it into four parts so that the maximum width of each strip was identical. These strips were then arranged in new, sterile Petri dishes to create an arena with four regions featuring gradually increasing doses of the above substances. This arena was then covered with a thin layer of agar-agar (#1.1614, Merck, Darmstadt, Germany) at 0.3% [*w*/*w*] and allowed to cool for another hour. At the beginning of the experiment, *M. morganii* was suspended in 0.9% NaCl at an OD_600_ of 0.2, and 10 µL was carefully added into the thin agar above the lower end of the strip containing no bactericides. The plates were cultivated in a humid chamber at RT to prevent the upper layer from drying out and evaluated after 1, 2 and 3 days.

### 2.5. Laboratory Biofilm Assays

Bacterial strains were allowed to grow in tryptone soy broth (TSB; #1.05459; Merck, Darmstadt, Germany) at 30 g L^−1^ for up to 3 days with shaking at 80 rpm at RT. The samples were subsequently diluted in fresh TSB to an OD_600_ of 0.2 and allowed to rest for another hour at RT with shaking (80 rpm). Next, these cultures were diluted 1:1000 in the medium specific to the respective experiment and sown into the wells of a 96-well-plate biofilm inoculator (MBEC Assay^®^ Biofilm Inoculator with 96-well base and uncoated pegs, Innovotech, Edmonton, AB, Canada) (V = 180 µL). Controls contained the respective medium only. Species combinations were created directly in the wells by adjusting the volumes (90 µL + 90 µL and 60 µL + 60 µL + 60 µL, respectively). Plates were subsequently incubated for 2 days on a horizontal shaker at 60 rpm. Growth was determined by transferring the supernatant to a new 96-well tissue culture plate (#92697, TPP, Trasadingen, Switzerland) and measuring the OD_600_ of the supernatant on a GloMax Explorer microplate reader (Promega AG, Dübendorf, Switzerland). To test the effect of an ecology change, the peg plate was transferred to a new base plate that had previously been filled with the required test media (180 µL well^−1^) and incubation continued on a horizontal shaker (RT, 60 rpm) for the time indicated.

Biofilm biomass was determined by staining the peg plate in a new 96-well tissue culture plate filled with crystal violet (#1.09218, Merck, Darmstadt, Germany), diluted 1:100 in sterile tap water at 180 µL well^−1^ for 2 min. The peg plate was subsequently transferred to a new 96-well tissue culture plate containing 70% EtOH (180 µL well^−1^) and incubated for 10 min. on a horizontal shaker (60 rpm, RT). The biofilm biomass was determined by measuring the intensity of the dissolved crystal violet at 600 nm.

### 2.6. Assessment of Population Dynamics by qPCR and Metagenomics in MWF Sediments

The sediment samples originated from a workshop that had used a mineral-oil-based, bactericide- and amine-containing MWF. These sediments had remained in the tank after the emptying and disposal of the fluid. They were humidified with deionized water and stored closed, but not airtight, at RT for six months before being used for the experiment.

Eighty grams of sediment was densely packed to the bottom of uprightly posed culture flasks with re-closable lids (#90652, TPP, Trasadingen, Switzerland) and then covered with 40 mL of MWF M and MWF S, respectively. Incubation was performed at RT on an orbital shaker (80 rpm) for 2 and 4 weeks, respectively. Following incubation, the sediment was partitioned as exactly as possible into the upper (UH) and the lower half (LH) of the sediment layer. These samples were carefully homogenized by vortexing and manual stirring with a spatula in 50 mL Cellstar^®^ tubes (Greiner Bio-One VACUETTE, St. Gallen, Switzerland) before analysis. In parallel, samples of the overlaying MWF (liquid phase) were collected.

DNA was isolated from both sample types using the DNeasy PowerSoil Pro Kit (#47016, Qiagen AG, Hilden, Germany). Briefly, duplicates of 250 mg of sediment or 250 µL of liquid, respectively, were added to the PowerBead Pro tubes, supplemented with 800 µL of Solution CD1, and homogenized using an MP Biomedicals FastPrep^®^ 24 classic bead beating grinder (LucernaChem, Luzern, Switzerland) for 45 s. at a speed of 6.5. The isolation continued according to the manufacturer’s instructions, and the duplicates were pooled at the isolation’s end. Aliquots of the retrieved DNA were used for qPCR of total bacterial load using the primer pair Aer8f (5′-GATCATGGCTCAGATTGAACGC-3′) and Aer101r (5′-CCAGGCATTACTCACCCGTCCG-3′) (developed by BioSmart GmbH, Bern, Switzerland; ordered from Eurofins Genomics, Ebersberg, Germany) using the SsoAdvanced™ Universal SYBR^®^ Green Supermix (Bio-Rad, Hercules, CA, USA) on a CFX-96 deep well real-time system attached to a C1000 Touch™ Thermal Cycler (Bio-Rad, Marnes-la-Coquette, France). The cycling parameters were a 95 °C hold for 180 s for initial denaturation and activation of the hot-start polymerase, followed by 40 cycles of annealing for 30 s at 62 °C, amplification for 30 s at 72 °C, and denaturation for 30 s at 95 °C. Fluorescence was read at the end of each amplification cycle. At the very end, a melting curve was conducted between 55 °C and 95 °C with a 0.5 °C increment read (5 s). Aliquots from the same DNA isolations were sent to Microsynth AG (Balgach, Switzerland) for amplicon metagenomics analysis using Microsynth’s standard primer set, including bioinformatics.

### 2.7. Alkanolamine Assays

Via our extensive customer service network, we had worldwide access to MWF from end users, both our own and external customers. We sourced MWF samples from a total of five workshops that had shown stability issues due to the loss of MIPA. These samples were pooled, and the microbial population was isolated by centrifugation at 12,000× *g* for 15 min at 15 °C in 2 mL centrifuge tubes (#0030 123.344; Eppendorf, Hamburg, Germany). The retrieved pellets were washed in 0.9% NaCl and once more pelleted by centrifugation at 12,000× *g* for 15 min at 15 °C. The resulting pellets were resuspended in MWF M in the same volume as the initial volume of the pooled samples and used for biofilm assays: aliquots (5 mL well^−1^) were sown into the wells of a 6-well tissue culture plate (#92006, TPP, Trasadingen, Switzerland), and the plate was incubated under static conditions for 1 week at RT. Then, the supernatant was carefully removed and the biofilms overlaid with 5 mL of freshly prepared MWF M supplemented with MIPA and TEA as described in Section 2.3. Incubation remained static and continued for another three weeks at RT.

For control experiments, microbial populations were retrieved as described above but then directly resuspended in MWF M supplemented with MIPA and TEA (initial volume). Five milliliter aliquots were incubated in 15 mL centrifuge tubes (#430791; Corning, Reynosa, Mexico) on a tube roller (Phoenix Instruments RS-TR05; Faust Laborbedarf AG, Schaffhausen, Switzerland) for three weeks at RT. Tubes were exchanged every two days to prevent biofilm formation.

For alkanolamine analytics, samples were diluted 1:10 (*v*/*v*) in 2-Propanol (Supelco LiChrosolv^®^ #1.02781, Merck, Darmstadt, Germany) before performing analysis by ESI-MS equipped with an ion trap (Thermo Fisher Scientific, Pratteln, Switzerland) using a Kintex HILIC column (100 mm × 2.1 mm, 1.7 µm) fabricated by Phenomenex (#00D-4474-AN; Aschaffenburg, Germany). The gradient elution conditions are shown in Table 1.

## 3. Results

For laboratory-based biofilm experiments, we chose three bacterial species that had previously been described as common in MWFs [8] as well as equal parts mixtures of them: *Pseudomonas oleovorans*, *Pseudomonas aeruginosa*, and *Morganella morganii*. *P. oleovorans* is arguably the most abundant species in mineral oil-based MWFs [12], and *P. aeruginosa* was described as a potent biofilm former [26]. *M. morganii* was included because it could be easily isolated from in-use MWFs by heterotrophic plate counts and was described to have highly reliable swimming skills [27] that were needed in mini MEGA experiments.

### 3.1. Adaptation to Toxic MWF Ingredients

#### 3.1.1. Bactericide Toxicity

**Figure 1:**
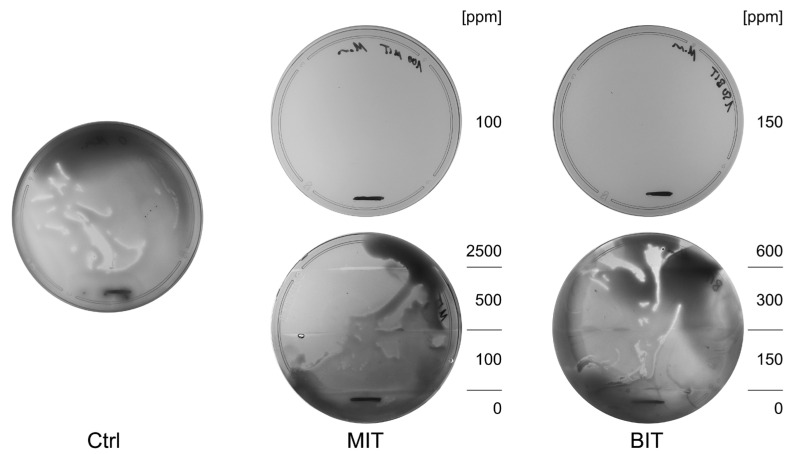
Resistance formation in real-time was tested over 3 days using assembled mini-MEGA assays: TSA plates contained increasing doses of MIT (0, 100 ppm, 500 ppm, 2500 ppm) or BIT (0, 150 ppm, 300 ppm, 600 ppm), whereas control experiments either contained no bactericides or the lowest dose of MIT and BIT, respectively. *M. morganii* appear as dark on the light background. Images were converted to grayscale and linearly contrast-enhanced but are otherwise unedited. A typical example of three independent experiments after an incubation of 3 days is shown. The visible lines in the lower part of the plates indicate the zone where bacteria were added.

Bactericides are an integral part of some MWF formulations to diminish the population size of planktonic bacteria. However, as it was shown that bacteria had the ability to survive even high antibiotic doses [28,29], we performed mini MEGA experiments in agar plates, inspired by the work of the Kishony Lab [30], and tested for possible adaptation of *M. morganii* to MIT or BIT. Direct exposure to the lowest dose applied completely suppressed growth and spread, which became perfectly feasible for subpopulations when exposure was gradual (Figure 1). This meant that bactericides were only effective when the organisms were directly exposed to them.

**Figure 2:**
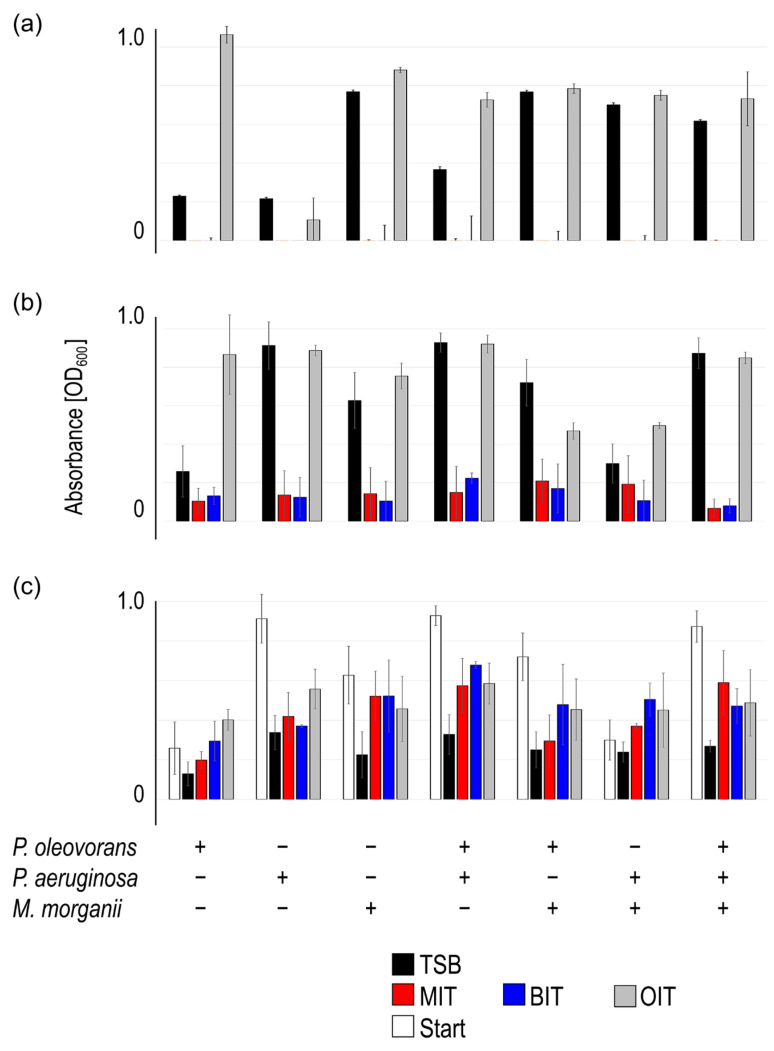
The impact of the biocides MIT, BIT, and OIT on consortia made up of *P*. *oleovorans*, *P. aeruginosa*, *M. morganii* and equal parts mixtures regarding planktonic growth (**a**), biofilm formation (**b**) and biofilm persistence (**c**). The mean value and standard deviation of three independent experiments are shown (obtained value minus control value without bacteria). Plus sign (+) indicates inclusion, minus sign (−) indicates exclusion of the respective species.

Bactericides are also added to MWF systems with the ulterior hope to have a measurable impact on the formation and destruction of biofilms, although it was previously shown that it does not work sufficiently [21]. Nevertheless, we constructed an experiment to test the effect of MIT and BIT on planktonic bacteria, the formation of biofilms, and pre-established biofilms of *P. oleovorans*, *P. aeruginosa*, and *M. morganii* and equal parts mixtures in the same set-up. We chose TSB as the background, which provided suitable growth conditions, and selected the fungicide OIT as a control agent. Growth of planktonic bacteria was successfully prevented by MIT and BIT, but not OIT, after 2 days (Figure 2a); however, biofilm formation was not (Figure 2b), indicating that at least a subpopulation was able to dislocate to surfaces, escaping the applied toxicity. Based on the results above, we assumed that bacteria in biofilms would be largely protected from any bactericidal action. Thus, biofilms were allowed to form in TSB before being exposed to MIT, BIT, or OIT. At maximum, biofilm biomass was reduced but not eradicated (Figure 2c). To our surprise, biofilms resuspended in fresh TSB decreased in all the species or species combinations tested. This could indicate that a change in the ecology, regardless of its extent, already impairs the stability of existing biofilms. However, it remained unclear why the addition of biocides weakened this effect.

#### 3.1.2. MWF Toxicity

**Figure 3:**
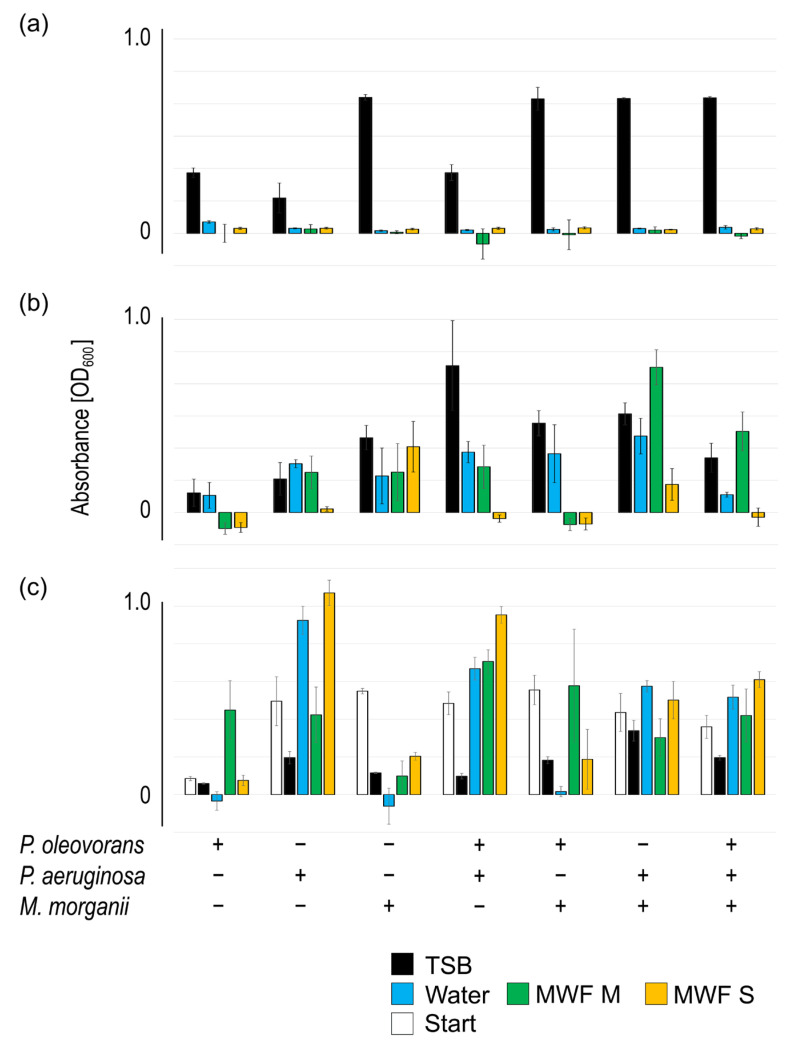
The impact of MWF M, MWF S, and tap water on consortia made up of *P. oleovorans*, *P. aeruginosa*, *M. morganii* and equal parts mixtures regarding planktonic growth (**a**), biofilm formation (**b**) and biofilm persistence (**c**). The mean value and standard deviation of three independent experiments are shown (obtained value minus control value without bacteria). Plus sign (+) indicates inclusion, minus sign (−) indicates exclusion of the respective species.

We and others [24,31] have found that bacteria grown up in TSB have difficulties persisting when cast into freshly prepared MWFs, especially at customary concentrations of 5% (*w*/*w*) or higher. This is likely due to the sudden change in environment, the presence of toxic ingredients, and the lack of readily metabolizable compounds. Even in the absence of listed bactericides [25], functional additives such as amines might be toxic to bacteria [32,33]. Along this line, we tested the growth capability of *P. oleovorans*, *P. aeruginosa*, and *M. morganii* alone or in equal parts mixtures in tap water, MWF M, MWF S, or TSB. Analogous to the experiments with bactericides above, growth after 2 days was basically only detectable in TSB (Figure 3a). Biofilm formation, however, still ensued in most cases (Figure 3b). In TSB and tap water, biofilm formation was measurable in all settings. In MWFs, however, *P. oleovorans* was unsuccessful and transmitted this behavior even in combination with the other two species, especially in the case of MWF S. *P. aeruginosa* and *M. morganii* formed biofilms to some degree and could even override the behavior of *P. oleovorans* in MWF M. This means that the outcome of inter-species interactions depends on the environment [31,34,35].

The time horizon of MWF systems, however, is several years, during which biofilm formation will inevitably occur. Thus, we let biofilms preform in TSB for 2 days and subsequently treated them with MWF M, MWF S, or water (Figure 3c). In numerous cases, the biofilm mass was effectively removed by water, while the other treatments were largely unsuccessful or even led to an increase. Especially biofilms containing *P. aeruginosa*, solely or in any of the combinations, remained unimpressed by subsequent treatments. This indicated that in established biofilms, some bacteria survived the environmental changes unscathed and even thrived and delineated themselves from it, analogous to experiments shown with bactericides (Figure 2).

### 3.2. Taxonomy Changes in MWF Sediments

Laboratory biofilms that start with the attachment of free-floating microbes to a clean or coated surface look remarkably different than those encountered in MWF systems. Here, microbial populations are interwoven with residues, sediments, and metal swarfs that are generated during production, float with the fluid, or stick to machine surfaces. Such sediments not only provide natural protection but also nutrients, as they contain precipitated organic substances from the MWF, tramp oils, and other sources [8]. We wanted to test the influence of freshly prepared MWFs on such consortia and obtained sediment samples from a machine tool that had been left behind after emptying (and disposing of) the mineral oil-based coolant.

Eighty grams of these sediments was densely packed to the bottom of culture flasks and overlaid with 40 mL of MWF M or MWF S. At the beginning of the experiment and after 2 and 4 weeks, respectively, we took samples from the upper (UH) and the lower half (LH) of the sediment layer and isolated the DNA. The total bacteria count varied little in both layers under all conditions as measured by qPCR. Thus, we switched to metagenomics and had the isolated DNA analyzed for its taxonomic structure up to the genus level.

**Figure 4:**
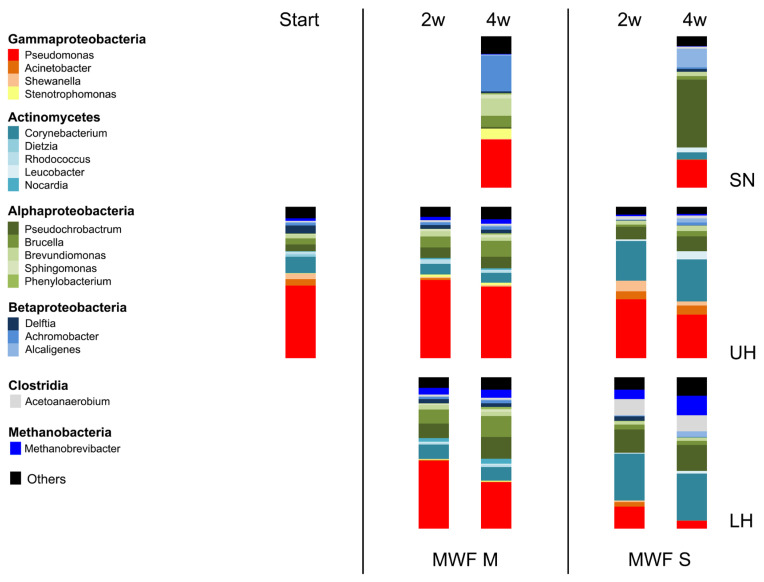
Bacterial populations at start and after up to 4 weeks of co-incubation with MWF M or MWF S in UH, LH, as well as the MWF itself (SN). Observed changes in MWF M were clearly less pronounced than in MWF S. The normalized mean value of two independent experiments is shown.

The sediments at the start of the experiment were largely dominated by Gammaproteobacteria, especially from the genus *Pseudomonas*. Incubation with MWF M up to 4 weeks changed the taxonomic structure, however on a small scale (Figure 4). Generally, the Jaccard Index [36] indicated that the population composition itself remained remarkably true to the initial situation and *Pseudomonas* was the dominant genus. However, in the LH after 4 weeks, there was an indication of change: Gammaproteobacteria, albeit still dominant, declined, whereas Alphaproteobacteria as well as Methanobacteria proportionally increased (Table 2). *Methanobrevibacter*, the only archaeal genus detected, had previously been discovered in mineral oil-based MWFs on a very small scale [12].

Diversity change was much more pronounced upon incubation with MWF S: *Pseudomonas* prominently decreased, whereas *Corynebacterium*, *Acetoanaerobium*, and *Methanobrevibacter* strongly increased (Figure 4), leading to a marked makeover of the population composition as indicated by the Jaccard Index (Table 2). Generally, MWF S led to a shift of dominance from Gammaproteobacteria to Actinomycetes, Alphaproteobacteria, Clostridia, and Methanobacteria, most distinctively in the LH. Remarkably, incubation with MWF S led to the loss of half of the detectable genera, which did not happen with MWF M. This showed that the microbial composition in sediments was altered depending on the MWF used, which could be due to increased competition [37]. The significance of this has yet to be determined.

Conversely, the environment also determined which taxa from the biofilm colonized it. And here both similarities and differences between the two MWFs became apparent. On the class level, both Actinomycetes and Methanobrevibacter were barely able to make the transition into both fluids, whereas Alphaproteobacteria and Betaproteobacteria were more successful than Gammaproteobacteria (Table 2). However, different genera were involved in the colonization observed in the two MWFs (Figure 4). What additionally surprised us was the fact that *Pseudomonas* was not able to maneuver itself into a dominant position. At least for MWFs based on mineral oil, such as MWF M, this contradicted earlier results [8]. This could be related to the experimental setup and the closed system or simply to the MWF chosen.

**Table 2:** Abundance of taxonomic units at start of the experiment and upon co-incubation with MWF M and MWF S, respectively, for up to 4 weeks. The normalized mean value of two independent experiments is shown. The Jaccard index [36] was used to calculate the similarity between the treated samples to the sediment at start.

MWF M	Start	UH 2w	UH 4w	LH 2w	LH 4w	SN 4w
Jaccard index	n.a.	97.3	95.5	95.1	74.5	59.9
Total genera	62	61	61	61	61	33
Gammaproteobacteria [%]	57.4	55.3	49.7	46.1	31.6	39.9
Actinomycetes [%]	14.5	11.0	9.6	13.7	14.6	0.7
Alphaproteobacteria [%]	11.6	19.2	23.0	23.0	34.3	24.6
Betaproteobacteria [%]	7.6	4.5	5.1	4.5	4.8	26.2
Clostridia [%]	0.7	1.3	1.2	1.2	1.3	0.0
Methanobrevibacter [%]	1.7	2.1	2.9	4.4	5.3	0.4
**MWF S**	**Start**	**UH** **2w**	**UH** **4w**	**LH** **2w**	**LH** **4w**	**SN** **4w**
Jaccard index	n.a.	84.9	69.8	39.1	20.6	29.8
Total genera	62	40	41	41	30	39
Gammaproteobacteria [%]	57.4	51.2	37.5	18.6	5.5	18.9
Actinomycetes [%]	14.5	27.3	33.1	31.8	32.8	9.0
Alphaproteobacteria [%]	11.6	12.5	17.0	20.9	22.1	50.0
Betaproteobacteria [%]	7.6	0.8	4.7	3.8	3.9	15.2
Clostridia [%]	0.7	2.1	1.9	10.7	10.6	1.4
Methanobrevibacter [%]	1.7	1.0	1.1	6.2	12.9	0.3

### 3.3. Functionality of Biofilms in MWF Systems: Alkanolamine Assays

Based on customer feedback, we found that the stability of some MWFs equipped with MIPA could be severely limited: After prolonged use, pH drops, stability and performance issues, as well as odor development and strong microbial colonization were reported. MIPA and other amines were popular ingredients of MWFs due to their functional properties, such as neutralizing acid-functional components and maintaining alkaline pH, as well as their alleged biocidal/biostatic tasks [33]. The search for the error finally led to the finding that MIPA was strongly or completely depleted, but not other alkanolamines such as TEA.

**Figure 5:**
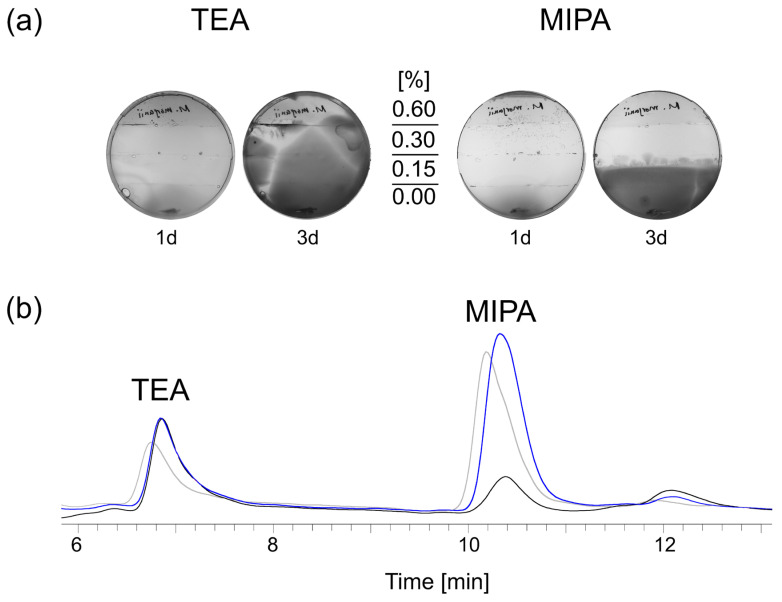
Resistance formation in real time was tested over 3 days using assembled mini MEGA assays: TSA plates contained increasing doses of TEA (0, 0.15%, 0.3%, 0.6%) or MIPA (0, 0.15%, 0.3%, 0.6%). *M. morganii* appear as dark on the light background. Images were converted to grayscale and linearly contrast-enhanced but are otherwise unedited. A typical example of three independent experiments is shown after 1 and 3 days. The visible line in the lower part of the plate indicates the zone where the bacteria were added (**a**). Biofilm (black), but not planktonic bacteria (blue) depleted MIPA after incubation for 3 weeks in supplemented MWF M, as shown by alkanolamine analytics. The untreated control is shown in grey. A typical example of three independent experiments is shown (**b**).

First, we wanted to test whether TEA or MIPA had adverse effects on *M. morganii* and carried out mini MEGA experiments. Within 3 days, survival and spread were unaffected at all doses of TEA, whereas MIPA already inhibited survival at medium doses (Figure 5a). From this, we concluded that the degradation of MIPA is due to the toxicity of the ingredient, making degradation the only viable option for thriving.

To test whether planktonic and/or biofilm populations were capable of degrading MIPA, we sourced MWF samples from workshops that had shown stability issues due to the loss of MIPA. These samples were pooled, and the microbial population was isolated by centrifugation. We subsequently employed these bacteria in planktonic form or allowed them to form biofilms in 6-well plates, respectively. Both populations were then incubated for 3 weeks with MWF M supplemented with TEA and MIPA before the remaining levels of these amines were determined by ESI-MS: A partial or complete loss of MIPA was detectable upon co-incubation with biofilms (Figure 5b), whereas TEA remained unaffected in all experimental set-ups. This signified that the degradation of MIPA was only possible from the protection of the biofilm layer. At that time, sorely, we only determined the composition of the used population by heterotrophic plate counts, which could not capture its diversity.

## 4. Discussion

In the metalworking industry, biofilms are widely regarded as a nuisance due to their detrimental effects known as biofouling or biodeterioration. Nevertheless, the focus routinely remains on the coolant itself and hardly on the countless square meters of surfaces hidden in the intricate structures of metalworking machines and plants [17,18,19], analogous to the problem with fungal contamination [14]. This is also reflected in the literature, which has only a few publications to offer in this regard.

The industry’s answer to the biofilm issue has long been the addition of bactericides, either as in-drum or tank-side additives. However, legal constraints to reduce the usage concentration of these chemicals are increasing [11], and our experiments showed that their effect on established biofilms was variable at best anyway. Importantly, adaptation to rather high concentrations of MIT and BIT, respectively, was easily possible—at least for *M. morganii*.

MWFs, on the other hand, are a complex mixture of a variety of organic compounds, and growth has been shown to be challenging for bacteria that enter the liquid unprepared [24,31], possibly due to the abrupt change in environment, which we were able to confirm. Importantly, however, biofilm formation still occurred, and the influence of MWFs on established biofilms was small to non-existent. This confirmed results from antibiotic research, which showed that biofilms are much more resistant than the corresponding planktonic bacteria [4].

Challenging to interpret were the results received with species combinations. Some, mainly those containing *P. aeruginosa*, seemed to work better than others or the respective single-species biofilms. However, there was no clear picture that certain combinations would always perform better in all the ecological environments tested. This leads us to conclude that the decision for or against cooperation depends on the actual environment, which may even change over time. This may indicate that results describing the social interactions of individual species in MWFs [31] were only correct in the respective context, while in another environment they could lead to completely different results. This might also have unpredictable effects on consortia that were tested for the biodegradation of spent MWFs [13].

When tests were continued using sediment biofilms, comparable results were obtained: The biomass itself, based on qPCR tests, remained stable, whereas the population composition shifted remarkably, depending on the MWF applied. Basically, MWF M led to fewer changes in population dynamics than MWF S did. One of the reasons might be, that the sediment used in those experiments originated from a MWF system running a mineral oil-based MWF. This might signify that distinct changes in MWF chemistry have a more pronounced influence on the population composition in biofilms than subtle variations, for good or ill. MWF S led to more pronounced increases of genera featuring more (facultative) anaerobes. This might have negative effects, as anaerobic species were described to be more important in microbiologically influenced corrosion [19]. On the other hand, strong environmental changes could also lead to a dispersion of biofilms [38], which could have a positive effect if the dissolved biofilm pieces were subsequently removed by filtration systems, which are often attached to machines or centralized systems [15,39]. Sorely, this was a scenario impossible to test within the experimental setup. In addition, our experiments revealed that the population composition in the liquid phase does not allow any conclusions to be drawn about the taxa in the biofilm phase: The population composition, although largely dependent on the biofilm input, was distinct. Likewise, it is impossible to draw conclusions about biofilm quantities [16].

The need to switch to biofilm analysis is underlined by the finding that these consortia are gaining function. In our case, this was the ability to degrade MIPA, which proved to be toxic to them. As this requires a large amount of biofilm to be present, it took a long time for noticeable symptoms to appear, such as a significant drop in pH, the appearance of odor, and the loss of technical properties. At least for the customers concerned, this all came out of nowhere. Interestingly, the bacteria in the MWF only became detectable by heterotrophic plate counts after the symptoms described had occurred. Even the addition of system cleaners and/or biocides and the removal of the fluid with subsequent refilling were rather ineffective, as the biofilms remained largely intact and quickly reformed again. Thus the “vicious circle” [17] started all over, often accelerating and resulting in a considerably shortened service life.

Overall, this condemns the microbiological analysis of coolant samples, as is common in industry today, to the status of occupational therapy. A switch to a methodology that focuses on the detection and characterization of biofilms is urgently needed and must be implemented in the coming years.

## Figures and Tables

**Table 1:** Gradient elution conditions. Phase A: 100% acetonitrile (#RC-ACNMS-2.5, Reuss-Chemie, Tägerig, Switzerland); phase B: 98.9% deionized water, 0.1% ammonium acetate (Supelco LiChropur™ #5.33004; Merck, Darmstadt, Germany) and 1.0% acetonitrile. The flow was 250 µL min^−1^.

Time [min]	Phase A [%]	Phase B [%]
0.00	95	5
5.00	70	30
10.00	70	30
20.00	50	50
22.00	95	5
25.00	95	5

## Data Availability

The original contributions presented in this study are included in the article. Further inquiries can be directed to the corresponding author.
